# Mesenchymal Stromal/Stem Cells in the Tumor Microenvironment and Their Role in Tumor Progression: Mesenchymal Stromal/Stem Cells and Tumor Progression

**DOI:** 10.31661/gmj.v11i.2637

**Published:** 2022-12-25

**Authors:** Venus Shahabi Raberi, Samira Abdollahi Moghadam, Ensieh Sharafi, Maryam Poudineh, Behnaz Barghgir, Maryam Molaee Eshgh Abad, Morteza Jafarinia, Hossein Kargar Jahromi

**Affiliations:** ^1^ Department of Cardiology, School of Medicine, Urmia University of Medical Sciences, Urmia, Iran; ^2^ Gynecologist and Obstetrician, Women's Reproductive Health Research Center, Faculty of Medicine, Tabriz University of Medical Sciences, Tabriz, Iran; ^3^ Department of Biology, Science and Research Branch, Islamic Azad University, Tehran, Iran; ^4^ School of Medicine, Mashhad Azad University, Mashhad, Iran; ^5^ School of Medicine, Shahrud University, Shahrud, Iran; ^6^ Shiraz Neuroscience Research Center, Shiraz University of Medical Sciences, Shiraz, Iran; ^7^ Research Center for Non-Communicable Disease, Jahrom University of Medical Sciences, Jahrom, Iran; ^8^ Zoonoses Research Center, Jahrom University of Medical Sciences, Jahrom, Iran

**Keywords:** Mesenchymal Stem Cells, Tumor, Cancer, Tumor Microenvironment, Mesenchymal Stromal Cells

## Abstract

Mesenchymal stromal/stem cells (MSCs) are a source of stem cells that can be easily harvested and differentiated into numerous cells. Over the past few decades, these cells have been introduced as promising therapeutic candidates for different diseases. Different studies have shown the role of these cells in regenerative medicine. Tumor growth is correlated with the interactions between MSCs and tumor cells in the tumor microenvironment. The precise key role played by MSCs in the progression of tumors is under question, and the effect of MSCs on the tumor is controversial it might involve the development of tumor initiation or prevent the spread of already existing ones. In this study, we reviewed the role of MSCs in the tumor microenvironment and their influence on promoting or inhibiting tumor progression.

## Introduction

In the past 20 years, few cells have received as much interest as mesenchymal stem or stromal cells (MSCs). MSCs are intriguing for a variety of physiological and pathological reasons, including their role in cancer, autoimmunity, organ transplantation, and tissue repair, as well as their enigmatic identity [1][2]. While the total effect of MSC appears to be primarily pro-tumorigenic, new studies on animal models reveal that MSCs could promote or restrict tumor growth. Today, several attempts have been made to develop new and safe methods for cancer treatment [3][4][5][6]. Nanotechnology and stem cell-based therapies are instances of novel approaches that drawing attention these days [7][8]. A tumor development, which initiates with stable chronic inflammation, weakened immunity, and tissue remodeling is referred to as a"wound that never heals" [9] Besides MSCs play a significant role in this process. The Immunomodulatory effects of MSCs affecting adaptive and innate immunity are important ways in which these cells influence tumor initiation and development. These immunomodulatory effects could be exerted by secreted substances, cell-cell interactions, or secreted exosomes [2]. The plasticity and properties of MSCs, such as pro-immunogenic, anti-inflammatory, and anti-tumorigenic effects, make these cells alluring therapeutic candidates in tumor therapy. It is also worth mentioning that the effects of MSCs on immune cells are opposing, as these cells may both promote inflammation or exert immunosuppressive effects, which cause the progression of tumors [10]. A detailed understanding of the interaction between tumor cells, MSCs, and immune cells is necessary, especially in light of how tumor cells can manipulate MSCs to work in their favor and the mechanisms underlying MSC plasticity that permit this to happen. In this study, we reviewed the role of MSCs in the tumor microenvironment and their role in promoting or inhibiting tumor progression.

## Mesenchymal stromal/stem cells

MSCs are mostly found in adipose tissue and bone marrow. They are a source of stem cells that are relatively easy to access, and can differentiate into numerous cell types, such as adipocytes, chondrocytes, and osteoblasts [11]. MSCs can be defined as plastic adherent cells that are positive CD73, CD90, and CD105, but do not display CD11b, CD14, CD19, CD34, CD45, CD79a, or HLA-DR [12]. According to Chen *et al*. (2006), MSCs have well-established roles in homing to injured tissues, suppressing innate and adaptive immune responses, and promoting angiogenesis. MSCs have a wide range of immune suppressive abilities, including stimulation of regulatory T cells (Treg), suppression of the activity of T-cells, modulation of the production of cytokine, and prevention of dendritic cell development [13]. Due to these characteristics, several research teams are now examining whether MSCs could be used to treat diseases associated with transplantation, such as graft versus host disease, autoimmune disorders, and, also targeted genetically engineered anticancer agents [14][15]. The capacity of MSCs to migrate to injured tissues has also been well-explained. The therapeutic effects of MSCs have been shown in the treatment of damaged kidneys, diabetes, bone injury, spinal cord injury, and myocardial infarction [12]. Tissue homing is linked to the production of different cytokines and chemokines, including Chemokine (C-C motif) ligand 8 (CCL8), CXCR2, CXCR1, CXCR4, matrix metalloproteinase-2 (MMP-2), tumor necrosis factor-alpha (TNF)-α, and stromal cell-derived factor 1 (SDF-1) [16][17]. In addition to immune suppression and tissue homing, MSCs can promote angiogenesis during ischemia and wound healing [18][19]. Together, MSCs exert several crucial features under physiological settings, and their special abilities have been employed to treat a variety of illnesses.

## MSCs and Metastasis

MSCs participate in several stages of tumor development. MSCs encourage the ability of tumor cells to invade and spread at the initial tumor location. Additionally, both human and mouse MSCs have been shown to promote the spread of breast cancer [20]. MSCs have been shown to go into tumor stroma and develop into cancer-associated fibroblast (CAF). Then, MSCs accelerated invasiveness, motility, and angiogenesis, while suppressing the apoptosis of cancer cells to promote colon cancer development and spread [21][22]. Additionally, it was discovered that in mice, CAFs moved from the initial tumor site to the lung metastatic site [23]. Similar to the way MSCs contributed to the spread of breast cancer, it was shown that hypoxia-inducible factors (HIFs) could transmit paracrine signals between breast cancer cells [24].

## MSCs and Epithelial-mesenchymal transition (EMT)

N-cadherin, TWIST, SNAIL, and vimentin levels were shown to increase when MSCs were co-cultured with gastric cancer or human breast cells, but E-cadherin levels were found to decrease [25][26]. Similar to this, human MSCs treated with TNF and interferon-gamma (IFN-γ) released higher levels of transforming Growth Factor-β (TGF-β). When Hepatocellular carcinoma cells were cultivated in conditioned media from IFN- and TNF-treated human MSCs significantly increased the invasion, migration, and expression of EMT markers in vitro and in vivo [27]. MSCs facilitated the spread of cancer cells to the lungs and bones via MSC-induced EMT [28]. Similarly, by enhancing the EMT process in MCF7 breast cancer cells, MSCs promoted the spread of breast cancer cells, whereas TGF-1 produced by MSCs enhanced EMT [29]. Additionally, MSCs were discovered to control EMT and tumor progression during the development of pancreatic cancer cells [30].

## MSCs and Tumor Microenvironment (TME)

In the tumor microenvironment, MSCs support the proliferation and spread of tumor cells. MSCs are connected to numerous stages of the etiology of cancer. During tumor progression, a significant number of MSCs generated from bone marrow were attracted to the tumor stroma [31].

### Immunomodulation

The immunomodulation effects of MSC in the tumor microenvironment have been proved by recent studies [32][33]. According to reports, MSCs possess immunosuppressive or immunomodulatory qualities [34]. Immunomodulation is thought to be mediated by cytokines released by MSCs, including TGF-β [35], IL-10 [36], nitric oxide (NO) [37], prostaglandin E2 (PGE2) [38], and indoleamine 2,3-dioxygenase (IDO) [39]. Previous studies have shown that the immunomodulatory capabilities of MSCs allow them to avoid being rejected by the host immune system [40][41]. MSCs' immunomodulatory abilities enable the treatment of a variety of inflammatory disorders [40]. Additionally, the rate of MSCs' immunological recognition influences the duration of their effects t [42]. A balance between the immunomodulatory components and the relative expression of immunogenic MSCs determines the rate of immune recognition and elimination of MSCs. Additionally, other reports have been published on MSC-related immunomodulation in tumor growth and progression. By promoting Treg cell activity, MSCs have been demonstrated to assist breast cancer cells [43]. TNF-α and IFN-γ stimulated the immunomodulatory activity of MSCs in melanoma. These cytokines promoted MSCs to express NO synthases [44]. It was discovered that the inflammatory cytokine IL-1α induces MSCs' immunomodulatory properties, allowing prostate cancer cells to evade immune surveillance [45].

### Angiogenesis

It was discovered that vascular endothelial growth factor (VEGF) expression by MSCs correlates to the angiogenesis of pancreatic cancer [46]. MSCs accelerate tumor growth in living organisms by enhancing the neovascularization around tumors [47]. Several soluble substances, including IFN-γ, TNF-α, VEGF, macrophage inflammatory protein 2 (MIP-2), leukemia inhibitory factor (LIF), and macrophage colony-stimulating factor (M-CSF), are secreted by MSCs to stimulate angiogenesis [48].

### Migration

EMT is a crucial step in the migration of cancer cells and can potentially promote carcinogenesis [49]. By causing cancer cells to separate from the initial tumor location, EMT encourages cancer cell migration and subsequent cancer cell spread [49][50]. MSCs in the TME promote tumor cell metastasis by inducing tumor cell EMT after being drawn to the tumor site. By co-culturing breast cancer cells and MSCs, the expression of SNAIL family members SNAIL (SNAI1) and SLUG (SNAI2) and vimentin increased [51], whereas E-cadherin expression decreased [52]. MSCs may have an impact on cancer cells through a variety of pathways, including the CXCR4 and estrogen receptor (ER) pathways in breast cancer [53], IL-6 and CCL5 [54], and CXCR2 [54]. By increasing MMP2 and MMP9 expression, MSCs also accelerate the invasion and migration of prostate cancer cells [55].

### Stemness

MSCs' multilineage capacity hastens the development of tumors. For example, MSCs altered the capacity of breast cancer stem cells to self-renew through cytokine networks, such as CXCL-7 and IL-6 [56]. MSCs associated with human ovarian cancer altered the synthesis of bone morphogenetic proteins to promote carcinogenesis [57]. Several signaling pathways, including those involving TGF-β, WNT [58], signal transducer and activator of transcription 3 (STAT3), Janus kinase 2 (JAK2), and IL-6, [59], in addition to bone morphogenetic protein signaling, increased the stemness of tumor cells. When MSCs are driven by cancer cells, they can establish a niche for cancer stem cells and cause carcinogenesis by producing a lot of PGE2 [60].

## MSCs inhibit cancer progression

Studies have shown that MSCs have an inhibitory effect on tumor growth in addition to their effects on cancer progression. The MSCs' interaction with tumor cells boosted the recruitment of granulocytes, monocytes, and T lymphocytes as proinflammatory agents. Increased infiltration of inflammatory cells promoted the opportunity for these immune cells to interact with the surrounding tissues. These immune cells, together with the nearby inflamed tissues, trigger anticancer immunity by producing several chemokines that induce the expression of appropriate chemokine receptors on T cells and their activation [61]. Additionally, MSCs were shown by Aarif and colleagues to reduce target cell AKT activity in Kaposi's sarcoma, which inhibited tumor growth in vivo. However, they found that Kaposi's sarcoma tumors were unresponsive to MSC injection when the Kaposi's sarcoma tumor cells were modified to consistently express active AKT. According to their research, MSCs effectively block AKT signaling to have anti-tumorigenic effects [62]. Similarly, Qiao *et al*. showed that MSCs block the Wnt pathway, which is essential for tumorigenesis, by suppressing breast cancer cell growth [63]. Additionally, Lu and colleagues demonstrated that the treatment of MSCs increased the expression of caspase 3 and p21 mRNA in tumor cells in their study. By inducing cancer cell apoptosis and G0/G1 phase stop, their results showed that MSCs can prevent cancer growth in vitro and in vivo [64]. Moreover, it has been demonstrated that MSCs can block tumor angiogenesis by causing endothelial cell death and capillaries deterioration [65]. Gu and colleagues recently revealed that a lncRNA C5orf66AS1/micro- RNA1273p/dual-specificity phosphatase 1 (DUSP1)/ERK axis was able to inhibit the malignancy of hepatocellular cancer stem cells (CSCs) [66]. Considering that exosomes play a role in the tumor-suppressing and oncogenic functions of MSCs, they treated hepatocellular CSCs with MSC-exosome and discovered that the CSCs' capacity for self-renewal, angiogenesis-stimulating, invasion, migration, and proliferation were significantly reduced through the lncRNA C5orf66AS1/microRNA1273p/DUSP1 axis and preventing the phosphorylation. Similar outcomes were observed in vivo, showing that exosomes slowed the xenograft growth created by CSCs in nude mice. Their research provides new perspectives on the significance of MSCs and the substances they produce for the advancement of cancer, particularly the CSCs stem cell quality. As modified, MSCs tend to move to tumor sites; they are widely populated. For instance, bone morphogenetic protein 4 (BMP4), nanoparticles, TNF-related apoptosis-inducing ligand (TRAIL), and other chemicals that can restrict cancer cell development were employed to change MSCs, which decreased cancer cell growth and metastasis while also inducing apoptosis [67][68]. These results suggested that migration and multiplication of cancer cells can be inhibited by modified MSCs, suggesting that MSCs may one day be used as a cancer treatment.

## MSCs promote cancer progression

MSCs, immunological cells, adipocytes, cancer-associated fibroblasts, and endothelial cells are only a few of the stromal cells found in TME [69]. MSCs in particular show a significant affinity for tumor sites, which can accelerate or arrest disease spread. However, the exact method is unknown. MSCs have Toll-like Receptors (TLRs), which are present in many different cell types. TLRs can recognize "danger" signals, and their activation draws a range of cells, including immune cells and MSCs, to the damaged area. Whereas TLR4 stimulation caused MSCs to produce proapoptotic and inflammatory factors (such as TRAIL, GM-CSF, and IL-17,), TLR3 activation caused certain factors with largely tumor-supportive immunosuppressive effects (such as IL10 and IL1RA). TLR4-primed MSCs, known as MSC1, showed anti-tumorigenic effects, whereas x TLR3-primed MSCs displayed a tumor-supportive effect [70]. Additionally, it has been shown that MSC1 causes a reduction in tumor growth while MSC2 promotes metastasis and tumor growth, according to Ruth and colleagues [71]. MSCs can transition between MSC1 and MSC2 depending on the used TLR agonist. In other words, the used agonist affects the polarization of MSCs. In this regard, studies have shown that TLR4 induces the polarization of MSCs into the MSC1, which is pro-inflammatory and is essential for early injury responses, but exposure to a TLR3 agonist induces the polarization of MSCs toward the immunosuppressive MSC2, which is required for assisting in the healing of tissue injury. It might aid in explaining why MSCs play a variety of roles in different cancer types. Also, MSCs interact with a variety of immune cells, including B cells, T cells, macrophages, dendritic cells, NK cells, and neutrophils, and secrete several mediators and soluble factors, including IL-1, IDO, IL-4, IFNs, and PGE2 [72]. It was also demonstrated that inhibiting the antitumor MSCs decreased the activation of T cells and proliferation during adaptive immunological responses. To rewire macrophages, MSCs release PGE2, which then binds to prostaglandin EP2 and EP4 receptors to cause the production of the IL-10, which is an anti-inflammatory cytokine, which in turn inhibits T cells [73]. MSCs also induced a Th2-polarized immune response. In this regard, anti-inflammatory Th2 cells and their related cytokines such as IL-4 increased while inflammatory Th1 cells and their related cytokines such as IFN-γ decreased [74]. Additionally, it has been demonstrated that MSCs inhibit the activation of T cells by secreting TGF-1 (an immunosuppressive cytokine), which binds to the glycoprotein a repetition predominant (GARP) produced on MSCs [75]. Furthermore, MSCs produce IDO which could inhibit allogeneic T-cell responses by decomposing tryptophan [39]. Notably, tryptophan catabolism sparked the emergence of Treg cells in CD4^+^ naive T cells [76]. By inhibiting effector T cell responses, these cells decreased anti-tumor immunity. Recent research has shown an entirely new way for MSCs to control the immune system. It is because MSCs recruit myeloid-derived suppressor cells (MDSCs) (inhibitory immune cells), which reduce anti-cancer T cell activity [77]. MSCs can inhibit B cell functions in the adaptive immune response in addition to T cell functions. By preventing B cell terminal development, humoral chemicals made by MSCs suppressed B cell activity [78]. Galectin-9 expression was enhanced by IFN-activated MSCs, which reduced the release of immunoglobulin upon antigen stimulation and decreased the proliferation of B cells [79]. When considered together, MSCs exert potent inhibitory effects on the adaptive immune response, which is heavily abused by cancer cells within TME. MSCs suppressed innate immune cells in addition to suppressing adaptive immune responses, weakening initial anti-cancer immune responses. MSCs' production of IL-6 and PGE2 inhibited NK cell activity. Additionally, MSCs mostly prevented NK cells from producing IFN-γ, which reduced their ability to fight cancer [80]. Furthermore, dendritic cells (DCs), which function to deliver antigens, are intimately associated with anti-cancer activity. It has been demonstrated that the presence of PGE2 produced by MSCs hindered the maturation and function of DCs [81]. Additionally, MSCs inhibited the growth and functionality of DCs produced from monocytes, with lower expression of the costimulatory markers CD80/CD86, hence restricting the ability of allogeneic T cells to also stimulate [82]. Moreover, MSCs directly decreased macrophage activity within the TME. It has been demonstrated that MSC-derived conditioned medium (CM) can decrease anti-cancer immunity by reducing the phagocytic activity of macrophages [83]. Elevated levels of IL-10 also induce MSCs to produce PGE2, which in turn causes the transition of M1 macrophages to M2 macrophages (a pro-tumorigenic state) [84]. Besides, MSCs had an impact on neutrophil activity. Co-culturing of MSCs with neutrophils could develop an immunosuppressive function in CD11b^+^ Ly6G^+^ neutrophils which in turn inhibit the proliferation of T cells and promotes tumor growth in a breast tumor model [85]. Similar to this, in gastric cancer, IL6-STAT3-ERK1/2 signaling controlled neutrophil chemotaxis, survival and activation, and promotes tumor development [86]. Together, the evidence presented above suggested that MSCs might suppress the anti-tumor immune response, which led to the development of tumors. Moreover, MSCs were able to promote angiogenesis and the proliferation of cancer cells. For instance, MSCs increased the levels of pro-angiogenic factors such as IL-6, TGF-β, VEGF, and MIP-2 in breast and prostate cancers. These elements accelerated the growth of solid tumors by promoting tumor cell proliferation and angiogenesis [87]. Likewise, Li *et al*. found that MSC treatment significantly decreased Smad7 mRNA expression while significantly increasing TGF-1 and microvessel density in hepatocellular cancer. Their research suggested that the TGF-1/Smad pathway may be used by MSCs to induce angiogenesis [88]. LncRNA H19 has recently been shown to be involved in MSC-mediated angiogenesis, according to Yuan *et al*. [89]. They discovered that LncRNA H19 knockdown in MSCs inhibited angiogenesis by interacting with histone methyltransferase EZH2 and activating the angiogenesis inhibitor gene VASH1, resulting in increased production of angiogenesis inhibitors and decreased secretion of angiogenesis factors. Additionally, MSCs accelerated the spread of cancer cells and hasten the growth of tumors. Co-culturing of MSCs with breast cancer cells could cause metastasis and significant overexpression of EMT-specific markers, proto-oncogenes (JUN, FYN), and oncogenes (FOS, NCOA4), and finally alterations in shape and growth pattern [25]. Notably, tumor metastasis is highly dependent on CSCs. According to evidence, MSCs produce various tumor-supportive mediators, which facilitate CSC proliferation and tumor progression [56]. The mesenchymal niche may also be involved in the spread of cancer. MSCs may be able to migrate to tumor locales, including primary and pre-metastatic sites, according to newly available information [90]. Tumor-secreted substances may reach nearby tissues [91], where they draw MSCs to aid in creating the mesenchymal niche, which promotes the migration of cancer cells. Breast cancer cells interact with CCR5 to increase cancer cell metastasis, invasion, and motility [92]. This causes MSCs to produce CCL5 (RANTES). MSCs could potentially stop tumor cells from going through the apoptotic process. As is well known, tumor development is influenced by hypoxia, starvation, and inflammation. MSCs maintain their survival through autophagy and the secretion of various anti-apoptotic molecules, including nitric oxide (NO), hepatocyte growth factor (HGF), SDF-1, TGF-α, basic fibroblast growth factor (bFGF), Platelet-derived growth factor (PDGF), and VEGF, [93]. B-cell lymphoma 2 (Bcl-2) expression can be increased by VEGF and bFGF, for instance, while TGF-β and PDGF can increase gene expression of bFGF and VEGF, respectively [94][95]. Leukemia cells have been demonstrated to be protected from spontaneous apoptosis by SDF-1 [96]. The angiogenic and anti-apoptotic effects were also enhanced by HGF [97]. NO was also believed to have a dual role in regulating apoptosis. Simply said, NO is proapoptotic at high levels but not at low doses. MSCs also promote tumor growth by altering their metabolic state. In lymphoblastic leukemia, MSCs-derived PGE2 stimulated cAMP-PKA signaling in tumor blasts and blocked wild-type p53's ability to prevent tumor growth, which encouraged leukemogenesis [98]. MSCs can generate lactate under oxidative stress in the TME, and when cancer cells take up lactate, they make ATP to aid in their migration [99]. MSCs, in particular have been seen to differentiate into CAFs in vitro, which may contribute to tumor heterogeneity and be essential for the development of cancer and drug resistance [100]. According to increasing evidence, noncoding RNAs are also implicated in drug resistance and cancer [101]. A recent study found that TGF-1 released by MSCs accelerated the growth of gastric cancer by activating the SMAD2/3 pathway and the MACC1-AS1/miR-145-5p/fatty acid oxidation (FAO) axis in cancer cells [102]. Additionally, MSCs dramatically stimulated the regulation of LINC01133 in nearby tumor cells in triple-negative breast cancer, which boosts the spread of CSC-like phenotypic traits and hence supports cancer cell development [103]. These results demonstrated that MSCs contribute to the development of cancer in many ways. A possible technique for the therapy of cancer is to target MSCs.

## Conclusion

The incredible diversity and plasticity of MSCs' involvement in tumor development are one of the most striking features of MSCs. Nearly every characteristic of cancer, including immune system evasion, pro-survival, anti-apoptosis, metastasis, and angiogenesis has been linked to MSCs. In vitro and mouse models, targeting MSCs as a component of anti-cancer therapy can considerably reduce tumor growth and metastasis and improve therapeutic features. There is a ton of information on MSCs' ability to promote tumor growth, but there is also evidence that MSCs can also slow the growth of tumors. Together, MSCs are critical modulators of therapy response and significant regulators of tumor growth. This makes these cells a desirable therapeutic target, deserving additional basic and translational research.

## Conflict of Interest

The authors declare no conflict of interest.
